# A Case Report on a Challenge From Diagnosis to Treatment: Interstitial Lung Disease As the First Manifestation of Systemic Sclerosis Sine Scleroderma

**DOI:** 10.7759/cureus.98714

**Published:** 2025-12-08

**Authors:** Susana Viana, Andreia Sá Lima, Verónica Guiomar, Inês Neves, Rui Môço

**Affiliations:** 1 Internal Medicine Department, Pedro Hispano Hospital, Matosinhos Local Health Unit, Matosinhos, PRT; 2 Pulmonology Department, Pedro Hispano Hospital, Matosinhos Local Health Unit, Matosinhos, PRT

**Keywords:** interstitial pulmonary fibrosis, mycophenolate mofetil (mmf), nintedanib, progressive interstitial lung disease, systemic sclerosis interstitial lung disease(ssc-ild), systemic sclerosis sine scleroderma (ssssc)

## Abstract

Interstitial lung disease comprises a heterogeneous group of conditions that may progress to pulmonary fibrosis. Establishing an accurate etiology is critical, as it dictates specific immunomodulatory therapy and profoundly influences prognosis. Systemic sclerosis is a rare autoimmune condition frequently complicated by interstitial lung disease, either during its course or, less commonly, as its initial or sole manifestation. Diagnosing systemic sclerosis in the absence of typical scleroderma features can be particularly challenging.

We present a rare case of pulmonary involvement as the initial manifestation of systemic sclerosis sine scleroderma. A man in his 70s presented to the Emergency Department due to gradually increasing dyspnea over the preceding two months. Upon admission, he was found to have hypoxemic respiratory failure. Thoracic computed tomography showed a fibrotic pulmonary pattern characterized by peripheral reticulation, honeycombing, and traction bronchiectasis. Bronchoscopy with bronchoalveolar lavage showed intense neutrophilic and mild eosinophilic alveolitis, along with increased alveolar macrophages. Initial clinical improvement was observed following treatment with corticosteroids and antibiotics, as infection could not be excluded. Following discharge and corticosteroid tapering, the patient's clinical condition worsened. Further immunological evaluation revealed positive antinuclear, anti-centromere, and RNA polymerase III antibodies. Pulmonary function tests demonstrated a mild restrictive pattern and a severely reduced diffusing capacity for carbon monoxide. In the absence of overt scleroderma or vasculopathy, a diagnosis of interstitial lung disease associated with systemic sclerosis sine scleroderma was established. Treatment with mycophenolate mofetil was initiated, and later combined with nintedanib, resulting in initial disease stabilization. Unfortunately, the patient succumbed to progressive and complicated pulmonary disease two years after initial presentation.

This case underscores the importance of maintaining a high index of suspicion when evaluating patients with interstitial lung disease, even in the absence of cutaneous manifestations of systemic sclerosis.

## Introduction

Interstitial lung disease (ILD) refers to a broad and heterogeneous group of diffuse parenchymal lung disorders that may progress to pulmonary fibrosis [1]. Although idiopathic pulmonary fibrosis is the most common form, approximately 15% of ILD cases are associated with an underlying connective tissue disease (CTD) [2,3]. Among CTDs, systemic sclerosis (SSc) is particularly relevant, as ILD represents one of its most common and severe organ manifestations, affecting up to 80-90% of patients radiologically and 30-50% clinically [4-6]. SSc-associated ILD is a major driver of morbidity and mortality, accounting for nearly one-third of SSc-related deaths [5,6].

A subset of patients of SSc, however, presents without clinically apparent cutaneous involvement - a phenotype termed systemic sclerosis sine scleroderma (ssSSc) [7]. Although rare, ssSSc poses a diagnostic challenge, as patients may initially lack the classical clinical clues that usually prompt investigation for SSc. In such cases, ILD may be the first or even the sole presenting feature, prolonging time to diagnosis and delaying appropriate treatment [7-9]. The progression from initial lung injury to life-threatening complications and death can be rapid and relentless [1,4]. This reality highlights the essential role of early detection, comprehensive management, and ongoing investigation into novel therapeutic strategies. Accurate determination of the underlying etiology of ILD is essential, as it directly impacts therapeutic decisions and prognosis [6,8].

In this report, we describe a case in which ILD was the first manifestation of ssSSc, emphasizing the diagnostic complexities and limitations encountered. This article was previously presented as a meeting abstract at the 22nd European Congress of Internal Medicine on March 8th, 2024.

## Case presentation

A man in his 70s, retired from the textile and locksmith industries and a former smoker, was admitted to the Emergency Department due to gradually increasing dyspnea and productive cough over the preceding two months. His medical history included diabetes, arterial hypertension, hyperlipidemia, ischemic heart disease, and cutaneous psoriasis, under inconsistent topical treatment. He also reported exposure to a pet canary and was up to date with COVID-19, influenza, and pneumococcal vaccinations.

**Figure 1:**
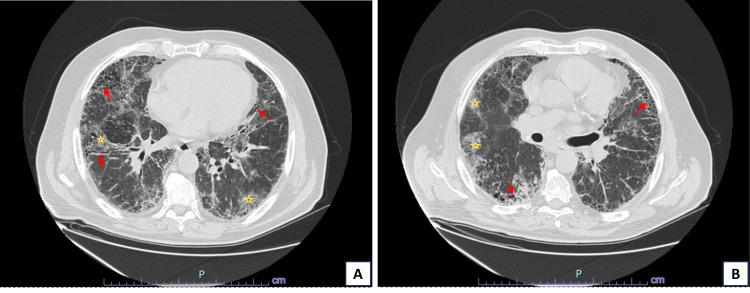
Axial images of initial chest CT in the emergency department. (A) and (B) show scattered bilateral subpleural reticulations, honeycombing, and traction bronchiectasis (red arrows), consistent with pulmonary fibrosis, along with ground-glass infiltrates (yellow stars).

On admission, the patient was febrile and tachypneic, with scattered pulmonary crackles on auscultation. No peripheral edema or other significant findings were noted on physical examination, apart from extensive psoriatic skin plaques. Laboratory results revealed slight hypoxemic respiratory failure and significantly increased C-reactive protein, with no other relevant abnormalities (Table 1). Thoracic computed tomography (CT) showed a fibrotic pulmonary pattern characterized by peripheral reticulation, honeycombing, and traction bronchiectasis, along with ground-glass opacities (Figure 1). Microbiological investigations, including respiratory viral panels, urinary antigens for Streptococcus pneumoniae and Legionella, and blood cultures, were negative. Bronchoscopy with bronchoalveolar lavage revealed no signs of malignancy but showed intense neutrophilic and mild eosinophilic alveolitis, with increased alveolar macrophages and no identifiable pathogens. Empirical treatment was initiated with corticosteroids (prednisolone 0.75 mg/kg) and broad-spectrum antibiotics with piperacillin/tazobactam (since infection could not be ruled out), and later switched to azithromycin following a positive Mycoplasma pneumoniae IgM serology. The patient demonstrated clinical and analytical improvement, enabling corticosteroid tapering.

**Table 1:** Evolution of analysis since hospital admission and over the years. *After antibiotic therapy and on prednisolone 60 mg/day **Off corticosteroid therapy and clinically worse. Corticosteroid therapy was restarted. ***On MMF 500 mg every 12 hours. **** On MMF 1 g every 12 hours (dose already reduced due to intolerance) and nintedanib 100 mg twice daily. The patient died about one month later. CRP: C-reactive protein. ESR: erythrocyte sedimentation rate. PCT: procalcitonin; MMF: mycophenolate mofetil

Parameters (units)	Admission	Discharge (10 days)*	Consultation (2.5 months)**	Consultation (1 year)***	Consultation (2 years)****	Normal Range
Hemoglobin (g/dL)	13.6	15	16.2	17.2	14.2	13-18
Leucocytes (^3/µL)	10.37	9.11	8.12	12.21	7.77	4-11
Platelets (^3/µL)	149	202	140	196	136	150-400
Creatinin (mg/dL)	1.1	0.9	0.9	1	0.7	0.7-1.3
CRP (mg/L)	241.9	2.9	<1	1.6	9	<5
PCT (ng/mL)	0.3	-	-	-	-	0.5
ESR (mm/h)	1	-	1	1	-	0-20

**Figure 2:**
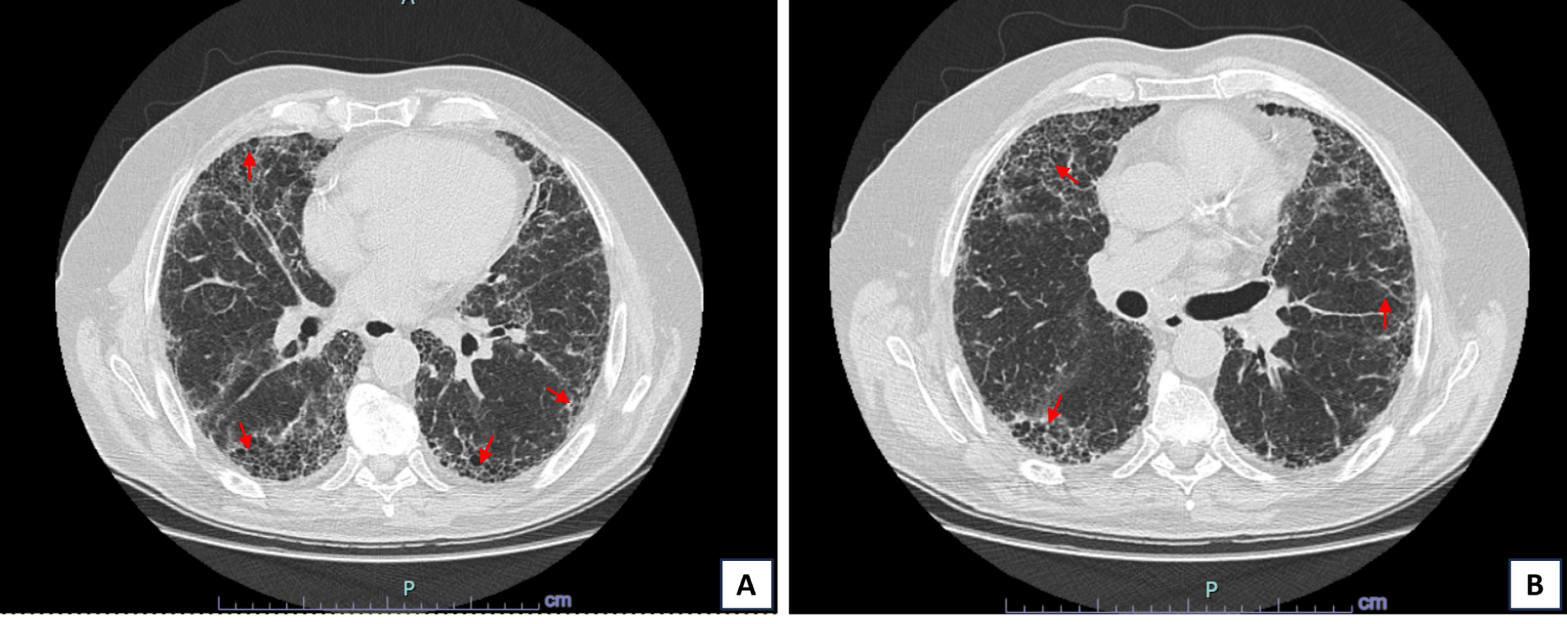
Axial images of six-month follow-up chest HRCT. (A) and (B) show resolution of ground-glass opacities compared with the previous CT. Presence of honeycombing, bronchiolectasis, and scattered traction bronchiectasis, suggestive of a fibrotic UIP pattern (red arrows). No areas of air trapping were observed. HRCT: high-resolution computed tomography

However, the patient experienced clinical deterioration a few months later. A follow-up chest CT revealed stable fibrotic changes with a definite usual interstitial pneumonia (UIP) pattern, clear resolution of ground-glass opacities, and no evidence of air trapping (Figure 2). Pulmonary function tests (PFTs) showed no restrictive pattern (Forced vital capacity - FVC 77%, > LLN), but a severely reduced diffusing capacity for carbon monoxide (DLCO, 30.7%). Further immunological workup showed high-titer antinuclear antibodies (ANA) and positivity for anti-centromere (ACA) A/B and anti-RNA polymerase III (anti-Pol-III) antibodies (Tables 2-3). Despite the absence of classic scleroderma features - such as sclerodactyly, puffy fingers, Raynaud’s phenomenon, or telangiectasias - a diagnosis of pulmonary involvement by ssSSc was established. Nailfold capillaroscopy showed nonspecific changes (capillary dilatations, tortuosities, and one microhemorrhage), and echocardiography revealed no signs of pulmonary arterial hypertension.

**Table 2:** Summary of immunological test results performed as part of the diagnostic workup. ACA: anticentromere antibodies; ACPA: anti-cyclic citrullinated peptide antibodies; ANA: antinuclear antibodies; anti-dsDNA: anti-double-stranded DNA antibodies; Anti-Ku: anti-Ku antibodies; Anti-MPO: anti-myeloperoxidase antibodies; Anti-PDGFR: anti-platelet-derived growth factor receptor antibodies; anti-PM-Scl75/100: anti-PM-Scl75/100 antibodies; Anti-Pol-III: anti-RNA polymerase III antibodies; anti-PR3: anti-proteinase 3 antibodies; anti-Ro52: anti–Ro52 antibodies; anti-Scl70: anti-topoisomerase I antibodies; anti-Th/To: anti-Th/To antibodies; anti-Nor90: anti-Nor90 antibodies.

Parameters	Result	Parameters	Result
ANA	1:1280	Anti-PM-Scl75/100	Negative
Anti-ACA A/B	Positive	Anti-Th/To	Negative
Anti-Pol-III	Positive	Anti-Nor90	Negative
Anti-Scl70	Negative	Anti-ds-DNA	Negative
Anti-Ro52	Negative	Anti-PR3	Negative
Anti-PDGFR	Negative	Anti-MPO	Negative
Anti-Ku	Negative	ACPA	Negative

**Table 3:** Summary of immunological test results performed as part of the diagnostic workup (continuation). C3: complement components 3; C4: complement components 4; Ig: Immunoglobulin

Parameters (Units)	Result	Normal Range
Rheumatoid factor (UI/mL)	<20	<30
IgG (mg/dL)	1319	540-1822
IgA (mg/dL)	323	101-645
IgM (mg/dL)	186	22-240
C3 (mg/dL)	114	82-185
C4 (mg/dL)	21	15-53

Treatment with mycophenolate mofetil (MMF) was initiated and titrated up to 3 g/day, followed by the addition of nintedanib, resulting in initial clinical stabilization and gradual corticosteroid tapering. Supplemental oxygen for exertion was prescribed for marked exertional hypoxemia.

**Figure 3:**
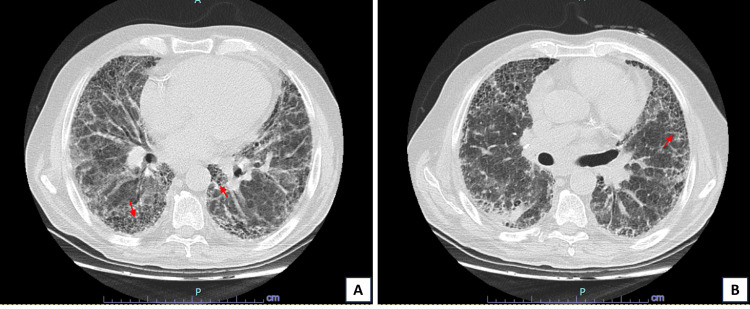
Axial images of chest CT two years later during hospitalization for an infectious complication. (A) and (B) with evident worsening of previously identified fibrotic changes (red arrows) indicative of advanced-stage pulmonary fibrosis.

Unfortunately, both MMF and nintedanib had to be dose-reduced due to gastrointestinal intolerance. After approximately one year of stability, the patient experienced worsening dyspnea and progression of pulmonary fibrosis (Figure 3), complicated by recurrent infections and repeated hospitalizations. This was accompanied by general health decline, progressive dyspepsia, and weight loss. The patient chose to discontinue further investigation, including endoscopic studies, and was referred to palliative care. He passed away shortly thereafter.

## Discussion

SSc is a rare and heterogeneous autoimmune disease characterized by small vessel vasculopathy, inflammation, and immune and fibroblast dysfunction, resulting in fibrosis of the skin and multiple internal organs [1,2,4-9]. ILD is a common complication, with 30-50% of patients developing clinically significant pulmonary involvement during the disease course [6,7,9]. However, interstitial changes can be detected on HRCT in up to 80-90% of cases [6,7]. The presence of ILD is associated with worse outcomes [4,6,7] and represents a major cause of morbidity and mortality in SSc, accounting for approximately 35% of SSc-related deaths [6,7,9,10].

SsSSc is a rare variant of SSc, defined by visceral involvement in the absence of the hallmark cutaneous manifestations [2,11]. As a result, patients may present with ILD before or without skin changes [3,11], making it challenging for clinicians to identify an underlying CTD in individuals presenting with ILD [2] and potentially delaying diagnosis. Therefore, SSc should be considered in all patients with pulmonary fibrosis [6,11]. Diagnostic tools such as ANA and extractable nuclear antigen (ENA) testing, esophagram, and nailfold capillaroscopy can assist in diagnosis [11]. Although there is no consensus on the optimal serologic tests for initial ILD evaluation, recent guidelines recommend ANA, anticyclic citrullinated peptide, and rheumatoid factor testing in all patients with suspected ILD, even in the absence of CTD features; and the ENA antibodies panel is advised when clinical suspicion is high, based on clinical ﬁndings or a positive ANA result [3,12].

Autoantibodies are detectable in over 90% of SSc patients [13]. The classical disease-specific autoantibodies include anti-topoisomerase (anti-Scl70), ACA, and anti-Pol-III antibodies, with specificities of up to 99.6%, 93%, and 97.5%, respectively [5,13-14]. Notably, our patient exhibited a rare and instructive serological profile with positivity for both ACA and anti-Pol-III. While ACA is typically linked to the limited cutaneous form and less severe ILD, and anti-Pol-III is strongly associated with scleroderma renal crisis and diffuse skin disease [1,4,6,9], their co-occurrence in a sine scleroderma patient with progressive ILD is highly unusual [2,3,11,15]. This underscores the heterogeneous nature of SSc and that antibody profiles may not always predict the phenotypic expression, particularly in ssSSc.

Most available evidence on ssSSc-associated ILD derives from small case reports or reviews [2,3,11,15], which consistently show ANA positivity [2,3,11,15]. Although data on other autoantibodies remain limited, several cases have also been reported with anti-Scl70 negativity [2,3]. Despite the absence of cutaneous involvement, most patients in these reports exhibited peripheral vascular involvement, such as telangiectasias, Raynaud’s phenomenon, abnormal nailfold capillaroscopy, or esophageal dysmotility [2,3]. However, some cases have been described without any additional findings [15], which may emerge years later, and not fulfill classification criteria in early stages [5], as occurred in this case. The development of specific diagnostic criteria for ssSSc would therefore be particularly valuable in such situations.

In the present case, the patient was a former smoker with occupational and bird exposure, which could predispose to ILD, such as pneumoconiosis or hypersensitivity pneumonitis. However, the radiologic features with absence of air trapping on HRCT, lack of lymphocytosis on bronchoalveolar lavage, and clinical deterioration despite corticosteroid therapy and exposure avoidance made these diagnoses less likely [16].

The UIP pattern is the second most common radiologic pattern in SSc, following nonspecific interstitial pneumonia [1,3,7]. The presence of intense neutrophilic and mild eosinophilic alveolitis with increased alveolar macrophages further supports the diagnosis of SSc-associated ILD (SSc-ILD) [6]. Lung biopsy could have provided additional diagnostic clarity, although it is rarely performed in the SSc-ILD context [4,6,17]. Small prospective and retrospective studies have reported associations between occupational exposures and more severe forms of SSc-ILD, suggesting a potential role in disease development [11,18].

The patient’s initial hospitalization appeared to be triggered by a respiratory infection in the context of pre-existing pulmonary fibrosis. Although no pathogen was isolated from bronchoalveolar lavage, Mycoplasma pneumoniae serology was positive, and the initial improvement was likely due to the antimicrobial therapy, with resolution of the ground-glass infiltrates (Figure 2).

The progression of SSc-ILD is highly variable. While some patients experience stable or limited pulmonary involvement, others undergo rapid and relentless fibrotic progression [4,8]. Several factors are associated with a more aggressive disease course of SSc-ILD, including older age at onset, shorter disease duration, presence of respiratory symptoms, reduced FVC, decreased DLCO, and the extensive interstitial changes on HRCT [4,7,9]. All of these factors were present in our patient. In this case, although the disease was temporarily stabilized with immunomodulation and antifibrotic therapy, the patient died two years after initial presentation due to disease progression. Gastrointestinal intolerance, the most common adverse effect of these medications [8,10], limited therapeutic options in this case.

The likelihood and risk factors for disease progression, including the extent of extrapulmonary involvement, should be carefully considered when initiating treatment. Immunosuppressive therapies for SSc-ILD have evolved significantly over the past decade [3,6], with a shift toward immunosuppressive and antifibrotic agents that have reduced reliance on corticosteroids, which should be used with caution due to the increased risk of renal crisis associated with high-dose prednisone use in SSc [3,6]. MMF, an inosine monophosphate dehydrogenase inhibitor, has emerged as a first-line therapy for SSc-ILD and diffuse cutaneous involvement, owing to its efficacy in treating both skin and pulmonary manifestations, its favorable side effect profile compared to cyclophosphamide in the Scleroderma Lung Trial II [19], and its safety when used in combination with other therapies such as nintedanib [8,9,17]. Nintedanib, an antifibrotic agent, has also demonstrated efficacy as adjunctive therapy in the SENSCIS trial, showing benefits in improving restrictive lung function in these patients [3,8,9,20]. Other treatment options for SSc-ILD include rituximab, cyclophosphamide, and tocilizumab [8,10,17]. Pulmonary transplantation and palliative care should be considered at all stages of the disease, and tailored to the individual patient’s needs [4].

## Conclusions

Pulmonary involvement as the initial manifestation of systemic sclerosis can be challenging to differentiate from other causes of ILD, often resulting in delayed or missed diagnoses. Early recognition is essential, as it significantly influences both prognosis and therapeutic strategy. This case represents a rare presentation of systemic sclerosis with isolated pulmonary involvement, associated with anti-centromere and anti-RNA polymerase III antibodies, and negative for anti-Scl70 antibodies. It is crucial to maintain a high index of suspicion when evaluating patients with ILD, even in the absence of cutaneous features of systemic sclerosis or in the presence of other potential contributing factors to pulmonary fibrosis. Therefore, a comprehensive autoimmune serological panel, including ANA, ENA, and specific SSc antibodies, is imperative in the diagnostic workup of any patient with fibrotic ILD, regardless of the presence of classic systemic features. This case highlights that the presence of multiple risk factors for progression (e.g., older age, low DLCO, UIP pattern) should prompt early and aggressive combination therapy, though tolerability often remains a limiting factor.
